# Independent and repeated acquisition of *Sodalis* endosymbiotic bacteria across the diversification of feather lice

**DOI:** 10.1098/rsos.251220

**Published:** 2025-09-17

**Authors:** Juliana Soto-Patiño, Kimberly K. O. Walden, Jorge Doña, Lorenzo Mario D'Alessio, Sarah E. Bush, Dale H. Clayton, Colin Dale, Kevin P. Johnson

**Affiliations:** ^1^ Illinois Natural History Survey, Prairie Research Institute, University of Illinois at Urbana-Champaign, Champaign, IL, USA; ^2^ Program in Ecology, Evolution, and Conservation Biology, University of Illinois at Urbana-Champaign, Urbana, IL, USA; ^3^ Roy J. Carver Biotechnology Center, University of Illinois at Urbana-Champaign, Urbana, IL, USA; ^4^ Departamento de Biología Animal, Universidad de Granada, Granada, Spain; ^5^ School of Biological Sciences, University of Utah, Salt Lake City, UT, USA

**Keywords:** Ischnocera, phylogenomics, Phthiraptera, avian lice, coevolution

## Abstract

Many parasitic insects, including lice, form close relationships with endosymbiotic bacteria that are crucial for their survival. In this study, we used genomic sequencing to investigate the distribution and evolutionary history of the bacterial genus *Sodalis* across a broad range of feather louse species spanning 140 genera. Phylogenomic analysis revealed significant diversity among *Sodalis* lineages in feather lice and robust evidence for their independent and repeated acquisition by different louse clades throughout their radiation. Among the 1020 louse genomes analysed, at least 22% contained *Sodalis*, distributed across 57 louse genera. Cophylogenetic analyses between the *Sodalis* and feather louse phylogenies indicated considerable mismatch. This phylogenetic incongruence between lice and *Sodalis*, along with the presence of distantly related *Sodalis* lineages in otherwise closely related louse species, strongly indicates repeated independent acquisition of this endosymbiont. Additionally, evidence of cospeciation among a few closely related louse species, coupled with frequent acquisition of these endosymbionts from free-living bacteria, further highlights the diverse evolutionary processes shaping *Sodalis* endosymbiosis in feather lice.

## 1. Introduction

Throughout evolutionary history, many insects have established associations with intracellular, heritable bacteria [1]. These endosymbionts often inhabit specialized host cells, providing benefits such as nutritional provisioning, enhanced digestion and protection against pathogens and environmental stresses [2,3]. These symbiotic associations not only benefit individual insects but can facilitate specialization of lineages into diverse dietary niches [4], ultimately shaping insect diversification [5]. Despite growing recognition of the significance of insect–endosymbiont associations in evolutionary research [6,7], significant gaps remain in our understanding. Specifically, patterns of evolutionary diversification of endosymbiotic bacteria and mechanisms of acquisition by insect hosts require further investigation. Such studies can offer valuable insights into the fundamental processes of host–symbiont coevolution.

The acquisition of novel endosymbionts in insect lineages is primarily explained by two models: host-switching (horizontal transfer from other insects) and acquisition from free-living bacteria [2,8,9]. Host-switching involves the transfer of endosymbiotic bacteria between host lineages, resulting in a phylogenetic tree topology in which the recipient lineage becomes united with the donor lineage on a long, well-supported branch [8]. Instances of host-switching by bacteria between insect lineages have been discovered in certain psyllids and aphids, where symbionts have independently colonized both unrelated and closely related insect hosts [8,10–12]. For example, Russell *et al.* [8] conducted a phylogenetic analysis of symbionts in aphids and psyllids, revealing well-supported clades within the bacterial sequences that united aphid symbionts with those of psyllids, suggesting horizontal transfer between these insect groups. This finding reveals that the genetic similarity of endosymbiotic bacteria across diverse hosts is the result of host-switching, highlighting the significant role of horizontal transfer in shaping symbiont distributions among both distantly and closely related insect lineages.

Alternatively, insects may acquire endosymbionts from free-living bacteria present in their environment. In this scenario, a large, genetically diverse bacterial population [13,14] colonizes an insect host and becomes an endosymbiont, sometimes replacing a pre-existing endosymbiont lineage [2]. Because free-living bacterium evolve slowly due to strong purifying selection, their acquisition by insects leads to an ancestral endosymbiont with a starting genome similar to this free-living ancestor, in a sense resetting the molecular evolutionary clock [15]. However, after transitioning to an endosymbiotic lifestyle, relaxed selection imposed on the maintenance of many genes, reduced effective population size and loss of DNA repair functions drive accelerated molecular evolution [14–16]. This accelerated rate can be detected by long branches of endosymbiont lineages compared to short branches of free-living lineages in a bacterial phylogenetic tree. Repeated acquisition of similar free-living bacteria across different insect lineages leads to a ‘star-like’ topology, with each lineage evolving independently from a similar ancestral state [15,17].

Documented examples of endosymbiont replacement or novel acquisitions span diverse insect groups, including aphids, psyllids, mealybugs, leafhoppers and feather lice [2,13,17–21]. Documented cases include both host-switching and replacement events in psyllids [12] and in parasitic feather lice, where distantly related bacterial genera have replaced earlier symbionts [15,17,22]. Given these patterns, lice provide an especially valuable system for examining the evolutionary drivers of endosymbiont acquisition and replacement.

Due to their relatively simple lifecycle and specialized dietary habits, parasitic lice (Insecta: Phthiraptera) offer an outstanding system for investigating endosymbiont acquisition. As permanent parasites of birds and mammals, lice complete their entire life cycle on the host, primarily transmitting by physical contact between hosts [23]. Notably, these lice display high host specificity, with the majority of louse species being specific to only one species of host [24,25]. Many species of lice have highly specialized diets, feeding exclusively on host blood or feathers, which lack essential vitamins for louse development [26,27]. Consequently, lice with such specialized diets depend on heritable endosymbiotic bacteria capable of synthesizing vitamins that are lacking in their diet [15,22,28–33]). Feather-feeding lice (Phthiraptera: Ischnocera) comprise over 3000 described species across over 150 genera [24,34]. Despite this diversity, little research has focused on the endosymbiotic bacteria that most feather lice seem to possess.

One bacterial genus that has been documented as an endosymbiont of feather lice is *Sodalis* [35]. Members of the genus *Sodalis* include both free-living species and insect endosymbionts, with endosymbionts found in a wide range of insect groups, including feather lice, stink bugs, mealybugs, psyllids, grain weevils and hippoboscid flies [3,36]. In feather lice, the *Sodalis* phylogeny from the dove-louse genus *Columbicola* is star-like, with weak internal node support and long terminal branches, a pattern indicative of repeated acquisitions from free-living ancestors. One such ancestor may have been similar to *Sodalis praecaptivus*, a free-living species of *Sodalis* with a notably short branch length in the phylogenetic tree [15,17], and endosymbionts with high identity (>98%) to *S. praecaptivus* are known [37]. Among the *Sodalis* endosymbionts from *Columbicola*, genome sizes range from around 0.9 to 3.1 Mbp [15], while *S. praecaptivus* has a genome size of 5.16 Mbp [38]. *Sodalis praecaptivus* was first isolated from a human hand wound caused by impalement on a tree branch [38,39]. Other free-living *Sodalis* species have also been described, such as *Sodalis ligni* found in decomposing wood [40]. Together, these free-living species represent plausible environmental sources for recurrent *Sodalis* acquisitions in feather lice. The biology of *S. praecaptivus* provides insight into endosymbiont acquisition, because the genus *Sodalis* is known for frequent transitions to endosymbiosis across insects, suggesting a predisposition to repeatedly colonize diverse hosts [38,39]. This versatility makes *Sodalis* a valuable model for studying the ecological pathways and evolutionary dynamics of endosymbiont establishment in insects.

To date, *Sodalis* endosymbiotic bacteria have been documented in a handful of genera of feather-feeding lice: dove lice (*Columbicola*), songbird lice (*Guimaraesiella*) and shorebird lice (*Carduiceps*, *Lunaceps*, *Quadraceps* and *Saemundssonia*) [15,17,41,42]. However, the distribution of *Sodalis* across the diversity of feather lice (Ischnocera) is unknown. Here, we employ genome-resolved metagenomic approaches to examine the presence of *Sodalis* across the diversity of feather lice, analysing data from over 1000 louse samples representing 140 feather louse genera. We use these data to reconstruct the phylogeny of *Sodalis* to understand the process of acquisition of these endosymbionts across the diversity of feather lice.

## 2. Methods

The workflow pipeline of this project (detailed below) leverages whole-genome sequencing, using metagenomic and phylogenomic techniques to resolve the cophylogenetic history of lice and their endosymbiotic bacteria.

### 2.1. Taxon sampling

Samples of 1020 chewing lice belonging to the parvorder Ischnocera [43] representing 140 feather louse genera (following classification of [24] with modifications of [44]) were selected for genomic sequencing or available from previously published data ([15,45–52]; electronic supplementary material, table S1). These previously published genomic data sets were generated for prior phylogenomic analysis of lice, and the current study leverages these raw reads to assemble the endosymbiont *Sodalis* genomes. These samples form the basis from which to explore whether *Sodalis* is present in a given louse species and then to build a phylogeny from the resulting *Sodalis* genome sequences that were obtained (below).

### 2.2. Genome sequencing

Genome sequencing, louse gene assembly and phylogenetic analysis follow the methods described in Johnson *et al*. [47]. The lice selected for genomic sequencing in this study were preserved in 95% ethanol and stored at −80°C. Individual lice were selected for extraction, and a photograph was taken and digitally deposited (see Data accessibility). Before extraction, individual lice were washed in a 1.5 ml vial of 100% ethanol. Total genomic DNA was extracted by first removing the louse from the vial and allowing the ethanol to evaporate. The louse specimen was then ground using a plastic pestle within a 1.5 ml tube. For the DNA extraction, a Qiagen QIAamp DNA Micro Kit (Qiagen, Valencia, CA, USA) was employed. The manufacturer’s protocol was followed, but modified by using an initial 48 h incubation at 55°C in tissue lysis buffer ATL containing proteinase K. The resulting purified and filtered DNA was finally eluted in 50 µl buffer AE. The quantification of the total DNA content was performed using a high-sensitivity kit with a Qubit 2.0 Fluorometer (Invitrogen, Carlsbad, CA, USA).

Genomic libraries were prepared using a Hyper library construction kit from Kapa Biosystems. The libraries were sequenced using Illumina NovaSeq 6000 with S4 reagents to obtain 150 bp paired-end reads. A set of dual-end adaptors was utilized for tagging the libraries, and they were multiplexed at 48 libraries per lane, with the aim of achieving approximately 30−60× coverage of the louse nuclear genome. These reads also typically contain similar coverage of the endosymbiont genome [15]. Lastly, adapters were trimmed, and files were demultiplexed using bcl2fastq v. 2.20, resulting in the generation of fastq files. For each library, the raw reads were deposited in NCBI SRA (electronic supplementary material, table S1).

### 2.3. Assembly and annotation of *Sodalis* sequences and phylogenomic analysis

The goal of this study was to reveal the distribution of *Sodalis* across the diversity of feather lice by analysing 1020 feather louse genomes (electronic supplementary material, table S1). Given the large diversity of samples to be analysed, we sought a method that could reliably detect *Sodalis* and provide a suite of genes for phylogenomic analysis in a reasonable timeframe. While de novo assembly methods can be highly successful (e.g. MetaWRAP using metaSPAdes [53]), they are often computationally intensive and produce extremely large results files (sometimes >100G–1Tb, pers. obs.), making these approaches not practically feasible for a study of this scale. Reference-based approaches are often more computationally efficient and can be quite successful in assembling genomes of the same genus as the specified reference. Therefore, we employed the reference-based assembly approach Mine Your Symbiont (MinYS) [54] to assemble the bacterial genomes, which allowed for comparatively rapid assembly times. The assembled contigs were annotated using the Microbial Genomes Atlas (MiGA) database [55], and tentative identification from this database was performed. MinYS uses a reference genome, in this case *S. praecaptivus*, to assemble a particular genome of interest from metagenomic data. This reference-guided assembler creates initial contigs from a subset of reads, which are then fine-tuned using all metagenomic reads in a de novo approach. The result is a genome graph that identifies strains with possible structural variations in the samples [54]. This approach performs well when the target genome is closely related to the reference [54]. Thus, given that we were specifically targeting *Sodalis* endosymbionts, this approach was ideal for the current study.

In the MinYS pipeline, more specifically, FASTQ reads from the louse sequencing libraries were mapped to the *S. praecaptivus* reference genome (NCBI: GCF_000517425.1) using the BWA aligner. Recruited reads were then assembled into contigs with the Minia short-read assembler in MinYS. Gaps between contigs were then filled with the genome-finishing mode of MindTheGap software [56]. The final pipeline step simplified the GFA-format assembly and converted it into FASTA output.

Following assembly, we annotated the assembled contigs using the MiGA database [55] to identify the closest available genomes and determine their taxonomic classification. The MiGA webserver allows the classification of unknown prokaryotic genome sequences based on the genome-aggregate average nucleotide and amino acid identity calculated against genomes available in two database options: ProK containing non-redundant complete and chromosomal-level assemblies in NCBI versus TypeMat containing type material from draft and complete genomes in NCBI. We analysed each set of MinYS-assembled contigs (minimum assembly sum >20 kb) against the more complete TypeMat database using the ‘Popgenome’ option.

To identify the presence of putative *Sodalis* contigs, we applied a conservative, quality-based classification framework centred primarily on genome completeness and annotation confidence. The most important criterion for *Sodalis* detection was the quality of the essential gene set (ESS file), as output by the MiGA pipeline, such that the sample could be included in a phylogenomic analysis. MiGA leverages a Ruby script from the enveomics collection [57] to identify 106 conserved ‘essential’ genes, which are typically single-copy and widely shared across Bacteria and Archaea. We required each *Sodalis* assembly to contain predictions for at least 55 of these 106 genes. Assemblies falling below this threshold were considered low-quality and excluded from downstream analyses. We also evaluated contamination scores provided by the TypeMat database in MiGA to assess assembly integrity and excluded assemblies with high contamination. As a supporting criterion, we used the MiGA RDP Classifier, which identifies 16S rRNA sequences and provides taxonomic assignments with confidence scores. We required a minimum 16S confidence score of ≥90%, although nearly all included detections exceeded 95%, ensuring contigs were confidently classified as *Sodalis*. Sequences of the recovered essential gene sets were used in phylogenetic reconstruction for *Sodalis* (see below).

To summarize the distribution of *Sodalis* detections across the diversity of feather lice, we first calculated, for each genus, the proportion of samples with confirmed *Sodalis* presence and absence, based on the MiGA outputs specified above. We also computed 95% Wilson confidence intervals for detection rates to account for uncertainty related to sampling effort and detection frequency. These proportions and confidence intervals were then visualized using a pruned genus-level phylogeny of Ischnocera, based on de Moya [58]. All graphics and visualizations were generated in R v. 4.4.1 [59].

### 2.4. Essential single-copy gene file processing, phylogenetic matrix preparation and tree inference

Based on the bacterial phylogeny of *Sodalis* and relatives in McCutcheon *et al*. [3], we selected published genomes of 30 species from 20 bacterial genera as the outgroups for the phylogenetic analysis, with the species *Pragia fontium* and *Budvicia aquatica* used to root the phylogenetic analysis (electronic supplementary material, table S4). These sequences were also annotated using MiGA to retrieve the same 106 essential gene set as for our novel *Sodalis* genomes. The nucleotide sequences for the essential genes were translated into amino acids using a custom Python script, and multiple alignments for each gene were produced with MAFFT v7.490 using the options ‘—auto, --preservecase, --adjustdirection, --amino’ [60,61]. The amino acid alignments were back-translated to nucleotide sequences with a custom Python script. Alignment gaps were trimmed using trimAl v1.4.rev15 setting the gap threshold to ‘gt 0.4’ [62]. Individual gene trees were constructed using IQ-TREE v2.1.3 (-m MFP) [63] and visualized in FigTree v1.4.4 [64] to identify any non-*Sodalis* sequences. Sequences that were clearly not *Sodalis*, such as those identified as *Burkholderia* in a few samples, were removed to prevent the inclusion of contaminated or chimeric data in further analyses. A concatenated gene set in FASTA format was then created using the AMAS concat function with a partitions file in Nexus format [65]. The final concatenated gene set tree was built in IQ-TREE v2.1.3 using the General Time Reversible (GTR) model, the Discrete Gamma model with four categories for rate heterogeneity and 1000 ultra-fast bootstrap replicates.

### 2.5. Gene assembly and phylogenomic analysis for lice

#### 2.5.1. Louse sequence assemblies and phylogenomic analysis

In our study, we sought to reconstruct the cophylogenetic relationships between *Sodalis* bacteria and their feather louse hosts. Thus, we also needed a tree for the lice that contained a *Sodalis* endosymbiont. To achieve this, we processed the raw genomic data from each feather louse sample that was determined to harbour *Sodalis*, assembled a set of ortholog genes and conducted a phylogenomic analysis following the methods described in Johnson *et al*. [47]. More specifically, raw reads were processed using fastp v0.20.1 for adapter trimming and quality control [66]. We used aTRAM 2.0 [67] to assemble 2395 single-copy orthologs from a reference set of protein-coding genes [43] from the human louse, *Pediculus humanus*. We translated the nucleotide sequences to amino acids using a custom Python script, and then we performed a phylogenomic analysis, first aligning amino acid sequences using MAFFT v7.471 [60,61] and then back-translating them to DNA sequences. The gene alignments were trimmed using trimAL v1.4. rev22 [62]. The resulting gene alignments were concatenated into a supermatrix using AMAS v1.0 [65]. For phylogenetic analysis, we employed IQ-TREE 2 v2.1.2 [63] with parameters for partitioning and model selection to reconstruct a tree based on the concatenated gene sequences. We rooted the tree using *Proechinophthirus fluctus* (Anoplura), a species with a published *Sodalis* endosymbiont genome [22]. Since Anoplura (sucking lice) and Ischnocera (feather lice) are closely related sister groups, this allowed us to focus on lice with the *Sodalis* endosymbiont for direct comparison with the evolutionary patterns of the *Sodalis* bacteria (above). Ultrafast bootstrapping with UFBoot2 was used to assess tree support [68,69]. To account for incomplete lineage sorting, individual gene trees were generated with IQ-TREE 2 (-m MFP) and used in a coalescent analysis to construct a species tree with ASTRAL-III [70]. This software also calculated local posterior probabilities for each node in the coalescent tree.

#### 2.5.2. Cophylogenetic analysis

We compared the partitioned concatenated louse and *Sodalis* endosymbiont trees using eMPRess v1.0 [71]. For this comparison, we pruned our overall bacterial tree to contain only *Sodalis* taxa from lice. This software summarizes events across equally parsimonious cophylogenetic reconstructions into median maximum parsimony reconstructions. We followed the cost scheme (duplication: 1, sorting: 1 and host-switching: 2) used in various published cophylogenetic studies on lice [45,47,72]. This cost scheme makes the combined weight of duplication and sorting equal to the weight assigned to host-switching, providing an alternative method for reconstructing conflicting nodes in parasite and bacterial trees. Notably, cospeciation is consistently assigned a zero cost in the techniques of cophylogenetic reconstruction. Because switching of endosymbionts between species of lice might be implausible, given that they are isolated on different bird host species, we also performed the eMPRess analysis to minimize host-switching events by setting the host-switching cost parameter to 15 (following [15]). The other cost parameters were left at the prior values (0 for cospeciation events, 1 for duplication and 1 for losses). To test whether the reconstructed cost was less than expected by chance, we randomized the *Sodalis* tree 100 times to compare the cost for the reconstruction of the actual trees to those from the randomized distribution. This essentially tests whether the *Sodalis* tree is more similar (i.e. contains more cospeciation events) than expected by chance. Additionally, we used the louse and bacteria phylogenies to build a tanglegram, using the R package phytools (v. 2.3-0) [73].

## 3. Results

### 3.1. Identification and distribution of *Sodalis* endosymbionts

The Illumina sequencing of genomic libraries derived from individual lice yielded a range of 19–110 million total 150 bp reads (Read 1 + Read 2) per sample. Using MinYS assembly with MiGA annotation, we obtained robust assemblies of *Sodalis* from 228 of the 1020 feather-feeding louse genomes analysed (22.35%; electronic supplementary material, table S1). These detections passed our conservative quality thresholds, including the recovery of at least 55 essential genes and low contamination scores. An additional 14 samples (only around 1.4% of total) exhibited high-confidence *Sodalis* detections based on 16S rRNA classification (≥90% confidence) but were excluded from downstream analyses due to failing genome completeness (<55 ESS genes) or high contamination levels. These excluded samples are highlighted in the electronic supplementary material, table S1. They belong to genera such as *Ardeicola*, *Brueelia*, *Guimaraesiella*, *Picicola* and *Priceiella*, all of which are also represented in the dataset by either confident positives or negatives, indicating that their exclusion likely did not bias genus-level patterns. A prior study of the louse genus *Penenirmus* [74] using de novo assembly methods (MetaWRAP) did not detect the presence of *Sodalis* across over 40 species of this genus, and this matched the results in the current study using the MinYS reference-based approach. In addition, other bacteria were detected among the assemblies with 16S rRNA classification, such as *Burkholderia* (likely not an endosymbiont), indicating that MinYS has the potential to assemble at least 16S from lineages highly divergent from the reference. Together, these results suggest that while we may not have detected every *Sodalis* present in the sampled lineages of feather lice, our results provide a general picture of the distribution and phylogeny of *Sodalis* across these insects.

**Figure 1.**
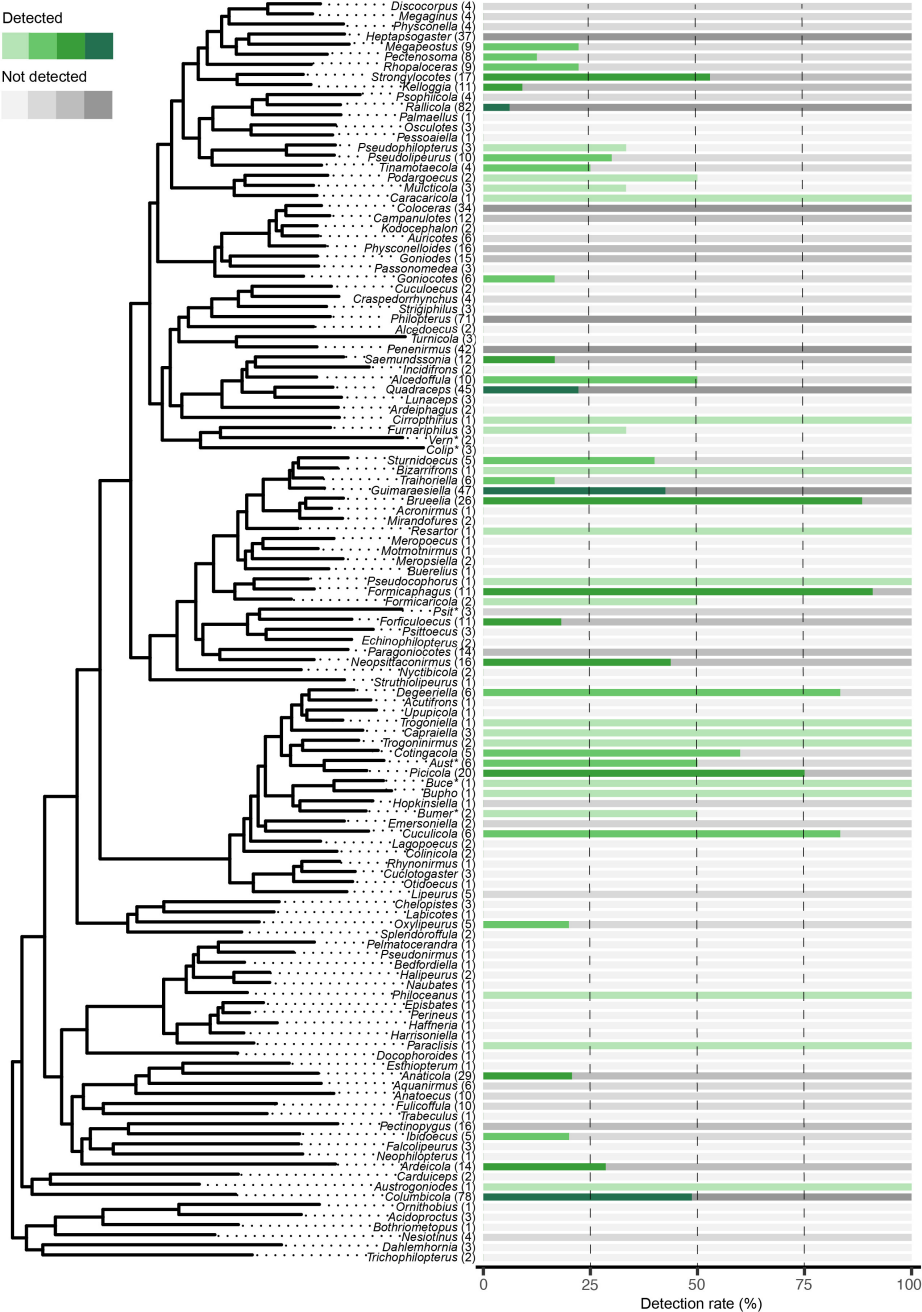
Genus-level phylogeny of Ischnocera (feather lice) pruned from de Moya [58], annotated with *Sodalis* detection patterns across genera. Horizontal bars to the right of each tip represent the proportion of samples in which *Sodalis* was detected (green) versus not detected (grey) per genus. Bar colour intensity reflects sample size: light (≤3), light-medium (4–10), medium-dark (11–29) and dark (≥30). Genus sample sizes are indicated in parentheses. Vertical dashed lines at the 25, 50 and 75% marks facilitate quick visual interpretation of detection rates. Detection rates and 95% confidence intervals are provided in the electronic supplementary material, table S2. Abbreviated genus names in the phylogeny refer to: Vern*, *Vernoniella*; Colp*, *Colilipeurus*; Psit*, *Psittaconirmus*; Aust*, *Austrophilopterus*; Buce*, *Buceronirmus*; Bupho*, *Bucerocophorus*; Bumer*, *Buceroemersonia*.

To explore broader genus-level patterns of *Sodalis* occurrence, we calculated the proportion of positive and negative detections for each of the 140 feather louse genera (electronic supplementary material, table S2). Among these, 57 genera included at least one sample with confirmed *Sodalis* presence. These detection rates were visualized on a pruned genus-level phylogeny of Ischnocera [58] to examine potential phylogenetic trends in prevalence (figure 1). Detection varied widely across genera, with some exhibiting consistently high prevalence (e.g. *Brueelia*, *Picicola*) and others showing very low (e.g. *Rallicola*) or no detections (e.g. *Penenirmus*, *Philopterus*). For example, among genera with more than 20 samples represented in the study, the prevalence of *Sodalis* ranged from 6 to 88%: 88.4% (23/26) in *Brueelia*, 75% (15/20) in *Picicola*, 20.6% (6/29) in *Anaticola*, 22.2% (10/45) in *Quadraceps*, 42.5% (20/47) in *Guimaraesiella*, 48.7% (38/78) in *Columbicola* and 6.2% (5/81) in *Rallicola*. Cases in which multiple individuals of the same louse species were sequenced generally indicated that these individuals harbour the same or near-identical lineages of *Sodalis* endosymbionts. For example, two individuals of *Columbicola tasmaniensis* (from two different dove hosts) had *Sodalis* endosymbionts differing by only 0.22% uncorrected pairwise sequence divergence across all ESS genes combined. Likewise, two individuals of *Strongylocotes* sp. from *Crypturellus soui* harboured *Sodalis* that differed by only 0.03%, and two individuals of *Saemundssonia wumisuzume* harboured *Sodalis* differing by only 0.21%. Some lice have genetically differentiated populations or cryptic species [75]. In many of these cases, the *Sodalis* from related louse individuals were closely related, yet genetically distinct. For example, samples of *Columbicola extinctus* from Band-tailed Pigeons (*Patagioenas fasciata*) in the US versus Peru harboured *Sodalis* endosymbionts that were sister taxa, yet genetically distinct (6.97% different). Similarly, cryptic species of dove lice, *Columbicola passerinae* 1 and 2, had related *Sodalis* species that were genetically differentiated (6.29%). Another example occurs between two individuals of the parrot louse *Neopsittaconirmus circumfasciatus* on *Alisterus chloropterus* and *Alisterus scapularis*, which had *Sodalis* differing by 4.06%.

### 3.2. Phylogenetic patterns in *Sodalis*

**Figure 2.**
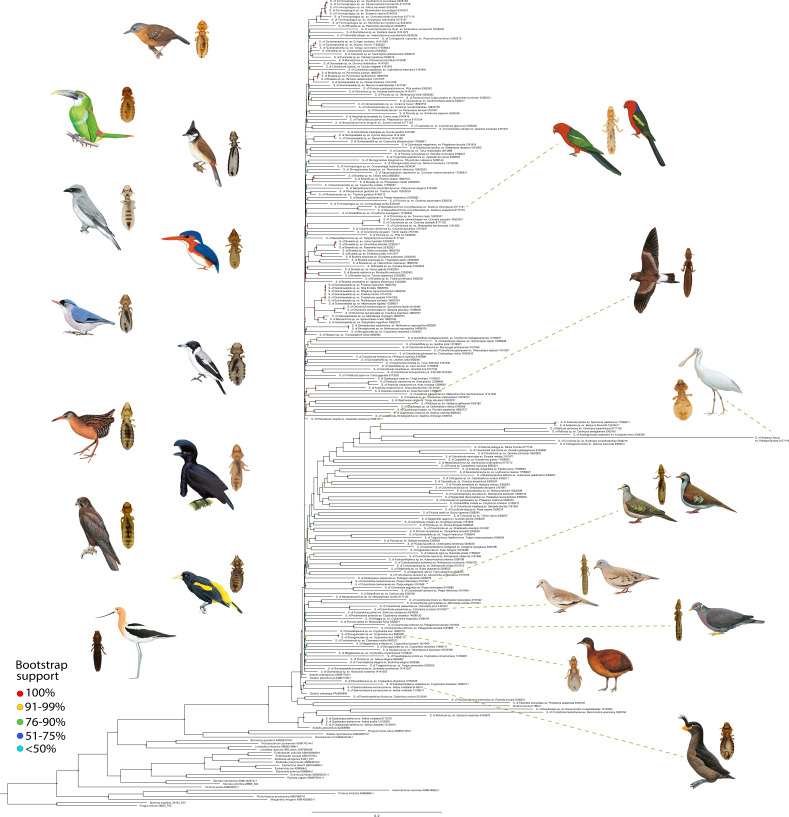
Phylogeny of *Sodalis* spp. symbionts from feather-feeding lice. Phylogeny of *Sodalis* spp. symbionts from feather lice (Ischnocera) and related bacteria based on a partitioned IQ-TREE ML analysis using the GTR model and the Discrete Gamma model with four categories for rate heterogeneity. Tree support assessed with 1000 ultra-fast bootstrap replicates. Values of bootstrap support on branches are indicated by colour bullets as follows: light blue (<50%), dark blue (51–75%), green (76–90%), yellow (91–99%) and red (100%). Branch lengths are proportional to substitution per site. Tips names indicate the *Sodalis* strain from each louse and avian host followed by respective SRA NCBI accession numbers. Bird and louse images on the left represent diverse examples of lice and their avian hosts from which *Sodalis* was identified. Bird and louse images on the right represent louse host of *Sodalis* that are mentioned in the text with lines connecting these lineages to associated images. Bird illustrations reprinted by permission from © Lynx Nature Books and © Cornell Lab of Ornithology (see electronic supplementary material, table S3 for licensing details).

The phylogeny resulting from IQTREE analyses of bacteria within Enterobacterales, including *Sodalis*, was generally very well resolved and supported in terms of relationships between bacterial genera (figure 2). However, the overall topology of the tree within the genus *Sodalis* reveals a star-like pattern. Specifically, the backbone of relationships among *Sodalis* endosymbionts of feather lice was characterized by short branches and low bootstrap support values. However, this phylogeny did reveal two main clades of *Sodalis*, each with 100% bootstrap support. Both of these clades included insect endosymbionts. Clade A included *Sodalis glossinidius*, the well-studied endosymbiont of the tsetse fly, along with *Sodalis* lineages from the louse genera *Quadraceps* (four species), *Mulcticola* (one species) and *Cirrophthirius* (one species). Clade B comprised the vast majority of feather louse-associated *Sodalis*. It also included *S. pierantonius* (a nascent/recently derived grain weevil symbiont) and the free-living *S. praecaptivus*, which are closely related to the majority of *Sodalis* strains from feather-feeding lice. In our analysis, we also included *Sodalis baculum* (a seed bug symbiont [76]), which is also a member of Clade B, clustering with other louse endosymbionts on a relatively long branch. In addition, *S. melophagi* (a sheep ked symbiont [77]) falls within Clade B, but on a relatively shorter branch, again clustering with other louse endosymbionts.

One notable feature of the phylogeny of *Sodalis* was the extreme variation in branch lengths. As in a prior analysis [15], the free-living *S. praecaptivus* was placed on a short terminal branch in comparison with endosymbiont lineages. However, some endosymbionts of lice were also on very short terminal branches. For example, *Sodalis* from *Philoceanus robertsi* was on a very short terminal branch, not markedly dissimilar to *S. praecaptivus*. In contrast, certain *Sodalis* lineages, such as the *Ibidoecus flavus* endosymbiont, were on very long branches, indeed, the longest branch in the entire phylogeny. Other *Sodalis* taxa in feather lice with the longest branches, including that of *I. flavus*, tended to cluster together. However, many of the nodes uniting these long-branch taxa had low bootstrap support (<90%), perhaps indicative of the artefact of long-branch attraction [78] or possibly arising from a common base compositional bias, often A + T bias, as documented in many symbiont lineages [79]. Although we did not analyse base composition bias across our dataset, previous work [15] has shown that in similar systems, taxa with longer branches tend to exhibit stronger A + T content. This pattern suggests that genome degeneration is accompanied by a GC : AT mutational bias, a common feature in insect symbionts.

### 3.3. Louse gene assembly and phylogenomic analysis

Assuming a genome size of 200−300 Mbp for Ischnocera [80,81], coverage of louse genomes ranged from around 20× to 100×. Assemblies of 2395 single-copy ortholog genes using aTRAM 2 [67] resulted in assemblies ranging from 872 to 2352 genes, depending on the sample, with an average of 2316 genes. After alignment, we retained 2376 genes for phylogenomic analysis. Following trimming, the concatenated alignment consisted of 3 857 202 aligned base positions. The analysis in IQ-TREE identified 432 optimal partitions with separate Maximum Likelihood (ML) models, producing a fully resolved tree with 100% bootstrap support for all but five branches, which were 98–99% (electronic supplementary material, figure S1). ASTRAL-III coalescent searches produced a nearly identical tree. The branching pattern of the main lineages of feather lice was generally identical to that of prior studies [45,82], albeit the taxon sample of the current tree was limited to those lice for which we found *Sodalis* as a likely endosymbiont.

### 3.4. Cophylogenetic analysis

When examining host distribution, the phylogenetic relationships of *Sodalis* across different feather louse genera show a mix of patterns. In several cases, some lineages of *Sodalis* from the same louse genus are closely related (e.g. *Anaticola*, some *Formicaphagus*). In many other cases, *Sodalis* from lice in the same genus are spread throughout the tree (e.g. *Columbicola*, *Strongylocotes*, *Brueelia*). These patterns suggest there could be a mix of codivergence between lice and *Sodalis* and phylogenetic incongruence.

We tested these patterns more formally by employing a cophylogenetic analysis. In particular, we aimed to assess the level of congruence between the host (louse) and endosymbiont trees. Generally, cophylogenetic reconstruction methods allow for cospeciation, host-switching, duplication and sorting events. For the cost scheme employed in many cophylogenetic studies in which cospeciation is 0, host-switching 2, duplication 1 and sorting events 1, the cophylogenetic reconstruction in eMPRess that included every sample as a terminal taxon reconstructed 73 cospeciation events, 0 duplications, 154 host-switches and 9 losses. The cost for this reconstruction is much less than that for random trees (*p* < 0.01), indicating more cospeciation events than expected by chance. Cophylogenetic reconstruction methods do not currently account for a scenario of repeated acquisition, so we repeated the analysis after increasing the cost of host-switching to 15, following Boyd *et al*. [15], to more closely simulate a scenario of repeated acquisition, while minimizing inferred host-switching. In this case, inferred duplication events and losses provide a measure of independent acquisition [15]. In this scenario, the reconstruction returned 125 cospeciation events, 91 duplications, 11 host-switches and 1113 losses, while congruence between the *Sodalis* and louse trees was still found to be significant (*p* < 0.01).

## 4. Discussion

Phylogenomic analysis of *Sodalis* endosymbionts of feather-feeding lice revealed significant diversity of *Sodalis* lineages and robust evidence for their independent and repeated acquisition by this group of insects. Among the 1020 louse genomes analysed, 22.35% contained evidence of associated *Sodalis* genomes, distributed across 57 genera of lice. The widespread, but not universal, nature of these endosymbiotic associations suggests *Sodalis* in feather lice have undergone multiple acquisitions and losses [83,84]. There are several lines of evidence [15] supporting this conclusion.

First, the Sodalis phylogeny shows features indicative of repeated acquisitions, including a star-like topology (detailed below) and marked incongruence with the feather louse phylogeny. In many cases, closely related lice harbour distantly related *Sodalis* lineages, producing a tangled cophylogenetic pattern. However, there is also evidence for consistency of the same bacterial lineage between individuals of a given louse species, as well as for shorter-term codivergence between closely related species of lice and their *Sodalis* endosymbionts. This supports previous findings from fluorescent *in situ* hybridization [35] studies that *Sodalis* in feather lice is maternally transmitted through the eggs, predicting a pattern of host–endosymbiont codiversification. In short, a mechanism of maternal transmission, together with the overall characteristics of the *Sodalis* phylogeny (below), provides evidence for repeated acquisition and replacement of *Sodalis* endosymbionts throughout the history of diversification of feather lice.

### 4.1. Star-like phylogeny

Our phylogenetic results indicate that the process of repeated acquisition of *Sodalis* by feather-feeding lice is occurring more broadly, not just within a few genera that have been previously studied [15,17,41,42]. One of the key findings of our study is the star-like phylogeny of *Sodalis* endosymbionts associated with feather lice, characterized by long terminal branches and very short internal nodes that are weakly supported. In addition, these *Sodalis* endosymbionts are very closely related to the free-living *S. praecaptivus*, which is on a very short terminal branch. Together, these patterns suggest that many independent acquisitions of *Sodalis* from a free-living ancestor have occurred throughout the radiation of feather-feeding lice [15,17]. This scenario of recurrent acquisitions is supported by previous studies demonstrating that free-living *Sodalis* have transitioned to an endosymbiotic lifestyle many times in distantly related insect hosts [3,15,36].

In the context of feather lice, the star-like topology indicates recurrent symbiont acquisition within a relatively short evolutionary timeframe, as suggested by evolutionary simulations [17]. In these simulations, a free-living ancestral bacterium transitions to endosymbiosis multiple times. In the free-living lineage, molecular evolution proceeds at a slower rate because a large effective population size allows natural selection to purge even very slightly deleterious mutations. In contrast, once a bacterial lineage becomes an endosymbiont, selection in the novel environment, combined with a much smaller effective population size within an individual host insect, results in an accelerated rate of molecular evolution, leading to long terminal branches for endosymbiont lineages. Many *Sodalis* endosymbionts have also lost mutation repair genes [15], which is expected to further accelerate their rate of mutation. These processes, together with the fact that symbiont genomes evolve in a strictly reductive manner, yield a scenario in which the symbiont gene inventories are subsets of their free-living progenitors, as evidenced by comparison with the extant, close free-living relative, *S. praecaptivus* [15]. Likewise, the multiple endosymbiont lineages derived from this ancestor do not fundamentally have a phylogenetic structure, yielding short, weakly supported internal branches.

Star-like phylogenies in diverse systems are often ascribed to dynamic evolutionary processes, such as frequent endosymbiont gains and losses. In various insect hosts, including weevils, stinkbugs and louse flies, *Sodalis* endosymbionts exhibit similar phylogenetic patterns characterized by long branches and weak internal node support, indicating independent acquisitions from environmental reservoirs [9,19,85]. For example, *Sodalis*-allied symbionts in *Sitophilus* weevils demonstrate a dynamic evolutionary history with frequent re-associations, acquisitions, horizontal transfers, replacements and losses [9,86]. In louse flies, the phylogenetic clustering and occasional replacements of *Sodalis* suggest multiple independent acquisitions over evolutionary time. Similarly, stinkbugs show variable *Sodalis* infection frequencies across species combined with host–symbiont phylogenetic incongruence [85]. These recurring acquisitions suggest that *Sodalis* bacteria have repeatedly transitioned between free-living and endosymbiotic lifestyles across diverse insect taxa, underscoring their adaptive versatility in establishing endosymbiotic relationships [36].

### 4.2. Phylogenetic and cophylogenetic relationships of* Sodalis* endosymbionts

Another indicator that repeated acquisition from a free-living ancestor may be occurring is that closely related louse species often harbour distantly related *Sodalis* strains. For example, some members of the louse genus *Quadraceps* harbour *Sodalis* endosymbionts from Clade A, while others harbour representatives from Clade B. This phylogenetic pattern suggests that these bacteria have been independently acquired by different louse species through multiple evolutionary events [17], leading to significant evolutionary divergence among endosymbionts even in closely related hosts. Within Clade B *Sodalis*, this pattern is also evident, with some louse genera (e.g. *Columbicola*, *Brueelia* and *Strongylocotes*) having *Sodalis* that appear in multiple positions throughout the phylogeny of Clade B. These patterns are reflected in widespread discordance between the louse and *Sodalis* trees. The origins of these acquisitions may trace back to different free-living progenitors, as *S. praecaptivus* is nested within Clade B with high support, while no free-living representatives have yet been identified in Clade A. This raises the possibility that Clade A and Clade B *Sodalis* arose from distinct ancestral bacterial strains that entered the feather louse system independently.

**Figure 3.**
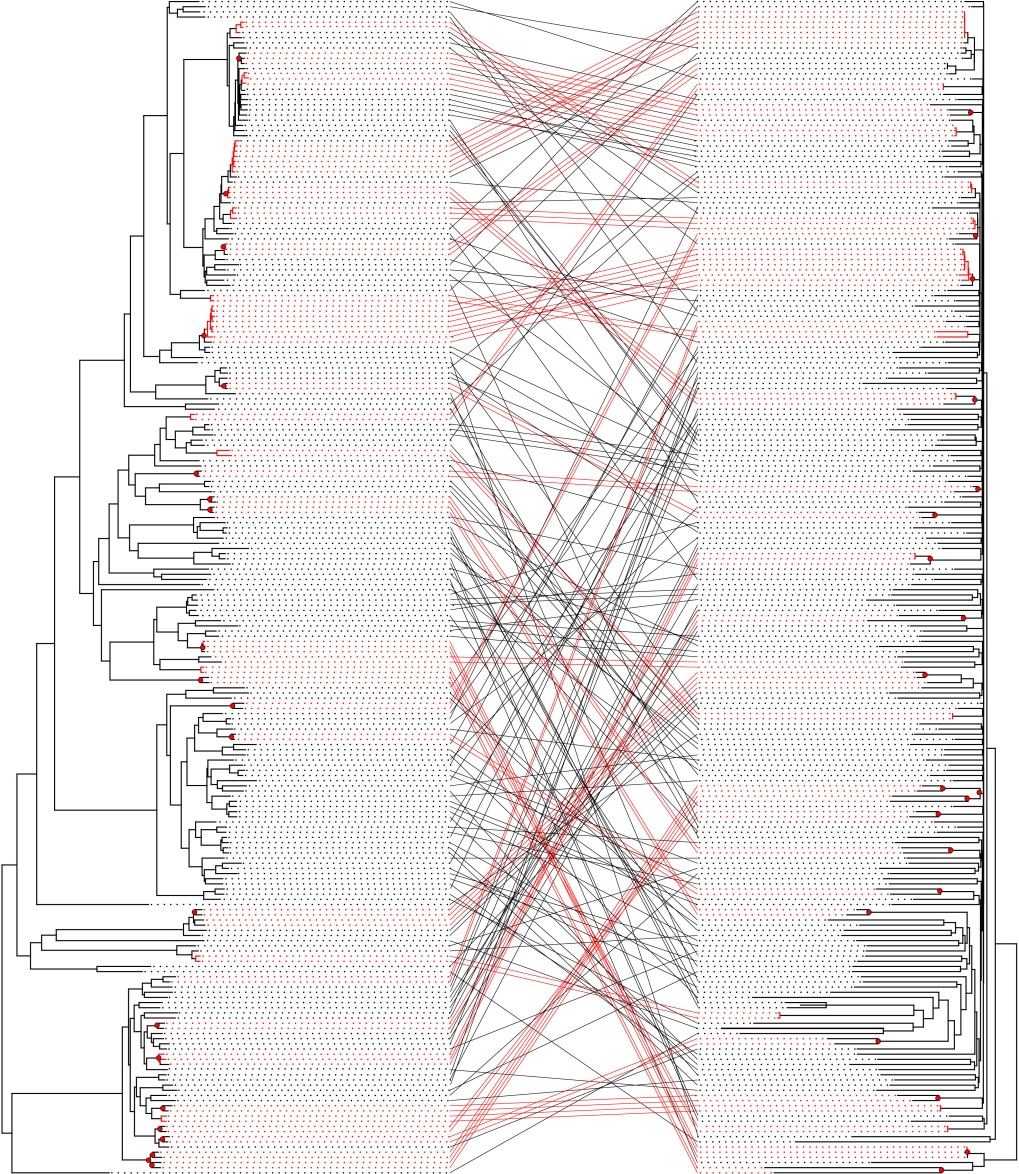
Tanglegram comparing the phylogeny of feather lice (left) with the phylogeny of their *Sodalis* endosymbionts (right). The louse tree was estimated from a partitioned IQ-TREE ML search of a concatenated matrix of 2359 single-copy ortholog genes (same orientation as electronic supplementary material, figure S1). The endosymbiont tree is the same as depicted in figure 2, but excludes the outgroups. Bulleted nodes connected with red lines indicate cospeciation events. However, in the case where the branches on the tree are also red, these represent the same louse species (based on COI sequences) and their associated endosymbiont.

Cophylogenetic comparisons of the louse and *Sodalis* trees revealed that the phylogenies of *Sodalis* endosymbionts and feather lice are largely incongruent. Vertical transmission of feather louse endosymbionts [17,35] would normally be expected to result in a pattern of widespread codivergence, where the phylogenies of the host and endosymbiont mirror each other. Such congruence has been observed in many other insect–endosymbiont systems, including psyllids and *Carsonella* [10], aphids and *Buchnera* [87], whiteflies and *Portiera* [88], weevils and *Nardonella* [89] and bat flies and *Aschnera* [90], among others. However, our cophylogenetic analysis of feather lice and their *Sodalis* endosymbionts revealed a relatively low number of cospeciation events (red dots and connecting lines, figure 3). These mainly occurred between very closely related terminal louse taxa or between cryptic species of lice, as has been found in other recent studies within single genera of feather lice [15,41]. Frequent replacement of endosymbionts is predicted to overwrite evidence of past louse-endosymbiont cospeciation events [15], leading to the overall patterns observed. Thus, while vertical transmission and cospeciation do occur, independent acquisition seems to be prevalent across the diversification of feather lice, especially over longer evolutionary timescales.

### 4.3. Host-switching

There are a few cases in the overall *Sodalis* phylogeny that are not consistent with either codivergence or a pattern of repeated acquisition from a free-living bacterial ancestor. These are cases involving *Sodalis* from somewhat distantly related louse hosts that are united on a comparatively longer, well-supported internal branch, which would be a phylogenetic pattern consistent with host-switching (horizontal transfer) of an endosymbiont from one louse lineage into another. One case occurs within the genus *Columbicola*, in which *Sodalis* from two species (*C. columbae* and *C. tsuschulysman*) are supported as sister taxa on a comparatively long, well-supported internal branch even though these lice are not closely related within the phylogeny of the louse genus *Columbicola* [91]. These two louse species occur on the same species of bird (the Rock Pigeon (*Columba livia*)), and this may provide an opportunity for an endosymbiont to switch from one louse host to another.

Another more complex case of potential horizontal transfer involves *Sodalis* in several species in multiple genera (*Guimaraesiella*, *Olivnirmus, Indoceoplanetes* and *Maculinirmus*) of lice within the *Brueelia*-complex. The *Sodalis* lineages (at least three) in these species are united by a long, well-supported internal branch. Unlike the case in *Columbicola*, these lice do not occur on the same species of bird, but do occur in the same general biogeographic region. Given that other *Sodalis* from some of these genera occur in other places in the *Sodalis* tree, it could be possible that these lineages represent a shared *Sodalis* ancestor in a common ancestor of the *Brueelia-*complex with subsequent loss and replacement. However, the phylogenetic relationships among the *Sodalis* lineages within this clade do not directly mirror the relationships of their louse hosts. Instead, this seems most likely to be a case of ancestral contact between ancestral louse lineages, resulting in the transfer of *Sodalis* from one louse lineage into others. Further investigation with additional sampling from these genera could be revealing as to the nature of this case. Overall, however, the phylogenetic pattern of the tree suggests that host switching of *Sodalis* between louse lineages is comparatively rare, if it occurs at all.

### 4.4. Variation in branch lengths

Although not necessarily directly related to the process of repeated acquisition, the significant variation in branch lengths within the *Sodalis* phylogeny reveals a dynamic evolutionary landscape for these endosymbionts. Terminal branch lengths vary by more than an order of magnitude across the tree. *Sodalis praecaptivus* occurs on a very short terminal branch, likely indicating the slow rate of molecular evolution within free-living bacteria that have very large effective population sizes. However, even within *Sodalis* endosymbionts of feather lice, there are some terminal branches that are remarkably short. For example, the *Sodalis* from *P. robertsi* is on a very short terminal branch, comparable to *S. praecaptivus*, indicating either a slow rate of molecular evolution or a very recent acquisition from a free-living ancestor. Most other *Sodalis* strains in feather lice, however, occur on much longer terminal branches, with the longest being *Sodalis* from *I. flavus*. This species forms a cluster together with many of the other longest branches in the tree, albeit with relatively low bootstrap support among these branches (<90%). This clustering may reflect the artefact of long-branch attraction [78] rather than any phylogenetic relationship. A further complication is that long-branch *Sodalis* strains also tend to have higher AT base composition [15], which may further cause long-branch taxa to cluster together. Overall, we posit that each of these long-branch taxa is an example of an independent acquisition event, and the application of phylogenetic analysis forces a tree structure among taxa, when in reality evolution did not proceed in a bifurcating fashion. Rather, independent origins from the same (or similar) free-living ancestor would produce the star-like phylogeny with variation in branch lengths that is observed in this study.

### 4.5. Future directions

Our study of *Sodalis* endosymbionts in diverse feather-feeding lice reveals promising directions for future research in evolutionary biology and symbiosis. We found strong evidence supporting independent and repeated acquisitions of these bacteria, posing several key questions for further exploration. Feather lice offer unique opportunities as a model to study repeated instances of the process of genome evolution across multiple endosymbiont acquisition events from related free-living bacteria [15]. The well-characterized genome of *S. praecaptivus*, which can be cultured, allows for detailed investigation into the consistent retention or loss of specific genes. Future comparative genomic analyses between the *Sodalis* endosymbionts identified in feather lice and other known *Sodalis* lineages, including free-living and insect-associated strains, would help clarify the extent and patterns of genome reduction, functional convergence and divergence across independent acquisition events. Such comparisons could also identify lineage-specific adaptations to different louse hosts.

Feather lice also offer opportunities to investigate evolutionary contingencies and mechanisms shaping genome evolution, such as shifts in base composition and accelerated mutation rates [15]. For example, recent work on *Sodalis* in *Columbicola* lice indicates that while genome degeneration is largely deterministic, stochastic processes can influence the loss of genes with redundant functions, producing patterns strongly shaped by historical contingency.

Another interesting question is why some groups of feather lice appear to have such a high prevalence of *Sodalis* endosymbionts, while others do not. Environment or geography may play a role in which bacteria are available for acquisition. However, many of the genera of lice included in our study are geographically widespread (like their avian hosts), and there is currently no clear pattern, beyond louse phylogeny, in the pattern of distribution of *Sodalis* across feather lice.

In conclusion, our study provides robust evidence for the independent and repeated acquisition of *Sodalis* endosymbionts in feather-feeding lice. By leveraging whole-genome sequencing and phylogenomic techniques, we have elucidated the distribution and evolutionary dynamics of these symbionts across diverse louse genera. Our findings contribute significant insights into the evolutionary patterns and mechanisms driving endosymbiont acquisition in insect-bacteria associations.

## Data Availability

Raw genomic reads for each sample have been deposited in NCBI SRA (electronic supplementary material, Table S1). Code and data for running analyses, including concatenated data matrix, gene alignments and gene trees, and all tree files are available from the FigShare digital repository https://figshare.com/s/9b914a733dbac506c7ef. Voucher lice photos are deposited in the Price Institute of Parasite Research, University of Utah, Salt Lake City, US (catalogue numbers: PIPR050302-PIPR051322; accessible via the ‘Symbiota Collections of Arthropods Network’ at https://scan-bugs.org and the ‘Ecdysis portal for live-data arthropod collections’ at https://ecdysis.org) and at the FigShare digital repository https://figshare.com/s/3ebae5aea796e59cc3be. This dataset includes high-resolution photos of the specimens analysed in the study. Each photo corresponds to a unique specimen identifier, providing visual documentation for verification and reference. Supplementary material is available online https://figshare.com/s/8e40c492c9a15cec3296.
